# From the track to the ocean: Using flow control to improve marine bio-logging tags for cetaceans

**DOI:** 10.1371/journal.pone.0170962

**Published:** 2017-02-14

**Authors:** Giovani Fiore, Erik Anderson, C. Spencer Garborg, Mark Murray, Mark Johnson, Michael J. Moore, Laurens Howle, K. Alex Shorter

**Affiliations:** 1 Department of Aerospace Engineering, University of Illinois, Urbana, Illinois, United States of America; 2 Department of Mechanical Engineering, Grove City College, Grove City, Pennsylvania, United States of America; 3 Mechanical Engineering Department, United States Naval Academy, Annapolis, Maryland, United States of America; 4 Scottish Oceans Institute, University of St. Andrews, St Andrews, United Kingdom; 5 Department of Bioscience, University of Aarhus, Aarhus, Denmark; 6 Biology Department, Woods Hole Oceanographic Institution, Woods Hole, Massachusetts, United States of America; 7 Pratt School of Engineering, Duke University, Durham, North Carolina, United States of America; 8 BelleQuant Engineering, PLLC, Mebane, NC, United States of America; 9 Department of Mechanical Engineering, University of Michigan, Ann Arbor, Michigan, United States of America; Universitat Zurich, SWITZERLAND

## Abstract

Bio-logging tags are an important tool for the study of cetaceans, but superficial tags inevitably increase hydrodynamic loading. Substantial forces can be generated by tags on fast-swimming animals, potentially affecting behavior and energetics or promoting early tag removal. Streamlined forms have been used to reduce loading, but these designs can accelerate flow over the top of the tag. This non-axisymmetric flow results in large lift forces (normal to the animal) that become the dominant force component at high speeds. In order to reduce lift and minimize total hydrodynamic loading this work presents a new tag design (Model A) that incorporates a hydrodynamic body, a channel to reduce fluid speed differences above and below the housing and wing to redirect flow to counter lift. Additionally, three derivatives of the Model A design were used to examine the contribution of individual flow control features to overall performance. Hydrodynamic loadings of four models were compared using computational fluid dynamics (CFD). The Model A design eliminated all lift force and generated up to ~30 N of downward force in simulated 6 m/s aligned flow. The simulations were validated using particle image velocimetry (PIV) to experimentally characterize the flow around the tag design. The results of these experiments confirm the trends predicted by the simulations and demonstrate the potential benefit of flow control elements for the reduction of tag induced forces on the animal.

## Introduction

Many marine mammals are efficient, agile and highly streamlined swimmers well-adapted for locomotion in the marine environment [1]. These animals are also difficult to observe in the wild because of the significant amount of time spent under water in their marine habitat. While some marine mammals (e.g. dolphins, sea lions, seals) have been kept and studied in captivity, the size and environmental requirements of most marine mammals prevent close study in captivity [2]. As such, bio-logging tags that collect sensor data directly from the animal are particularly important for the study of marine mammals in the wild. Advances in electronics, sensors, packaging and attachment methods for bio-logging tags are enabling the collection of behavioral, physiological, and environmental data from a range of animals in their natural habitats [3–5]. While tagging plays an important role in the study of many animals, the attachment of external devices is not benign, and hydrodynamic loading is of particular concern for aquatic animals [6–7]. Tag volume, shape, position and orientation with respect to the flow can significantly affect the loading imparted to the animal, potentially affecting behavior [8–9]. Therefore, careful design of the tag housing has great importance because of the potential to reduce the impact. Here we present a design (Model A) for a streamlined housing that incorporates flow control elements to reduce the net hydrodynamic load created by a tag. We then examine derivatives of the Model A design to better understand the contribution of individual features to the overall performance of the design.

The effect of loading created by a tag on a swimming animal can be difficult to quantify under realistic conditions. Experimental studies have demonstrated that instrumented dolphins experience higher drag loading from tags or may modify their behavior in response to a tag [2, 10]. However, controlled experiments with cetaceans are time consuming at best and impractical for many species. Moreover, it may be difficult to translate swimming performance directly into tag design changes that minimize fluid loading. Computer aided design (CAD) and computational fluid dynamics (CFD) tools provide an alternative that can be used to improve the understanding of fluid flow around the tag and inform hydrodynamic tag design [11–15]. These simulation tools allow several iterations of a design to be evaluated and compared relative to the initial design before investing in tag manufacture. Experimental validation of these simulations is an important consideration before using the results to predict the loading effect on a swimming animal. Controlled validation studies can be conducted in flumes, but these studies are further complicated by the fluid dynamics created by the combined animal/tag system [13].

The location of the tag, the size of the animal, and the speed of the combined system through the water all affect the state of the fluid moving around the tag. Although it may seem advisable for the tag to be positioned as close to the skin surface as possible to minimize the flow interface, the proximity to the animal's body surface can cause non-axisymmetric fluid flow where flow under the tag is more constrained than flow over the tag. This results in an increased lift force acting on the animal [16]. As swimming speeds increase, lift generated by the tag can dominate the net hydrodynamic force imparted to the animal [13]. This force is transferred to the animal’s skin through the tag’s point of attachment, and large forces could result in early detachment of the tag or even injury. For suction cup attachments, lift will increase the vacuum force at the attachment site and increase the risk of cup induced damage to the skin (i.e. barotrauma). As such, the performance benefits that result from reduced drag loading must be balanced with the additional lift forces that arise from the pressure difference created by non-axisymmetric flow.

The lift generated by a bluff body represents a well-known problem in aerodynamics, particularly in the automotive industry [17–18]. Bluff bodies are characterized by small aspect ratios, i.e. small length-to-height values. When moving through a fluid the geometry of a bluff (or blunt) body creates a relatively large disturbance to the surrounding fluid compared to that of a slender and streamlined body in the same conditions [19]. In the automotive industry, techniques have been successfully adopted to reduce the detrimental effects of (positive) lift acting on a vehicle. High performance race cars, for example, are capable of generating negative lift (downforce) by means of two main features: Venturi underbody channels and inverted wings. Shorter *et al*., (2014) presented a design that incorporated a hydrodynamic half body with an underbody channel to direct and accelerate flow under the tag to reduce flow asymmetries and the resulting pressure differentials on the tag. However, CFD simulations and experimental measurements were used to show that the design reduced drag loading, but generated large lift forces suggesting that there was no substantial net benefit from the channel. The authors concluded that while the channel did direct and accelerate flow under the tag, the fluid became detached from the channel walls negating the intended benefit of the feature. Additionally, the design presented in Shorter *et al*. used an underbody channel without additional features such as a complimentary wing, potentially limiting the performance of the system.

**Fig 1:**
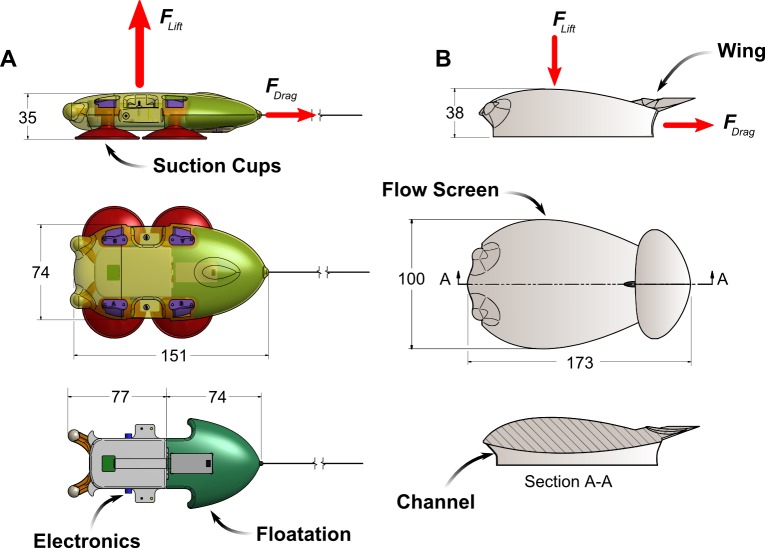
**(A)** A representative illustration of the Model 0 tag with a foil-like cross section. As swimming speed increases, the lift generated by this housing shape becomes the dominant force acting on the tag. The tag components that drive the packaging constraint are shown in the bottom left. **(B)** Illustration of a tag with a flow screen, wing and underbody channel designed to reduce the lift force acting on the tag.

**Fig 2:**
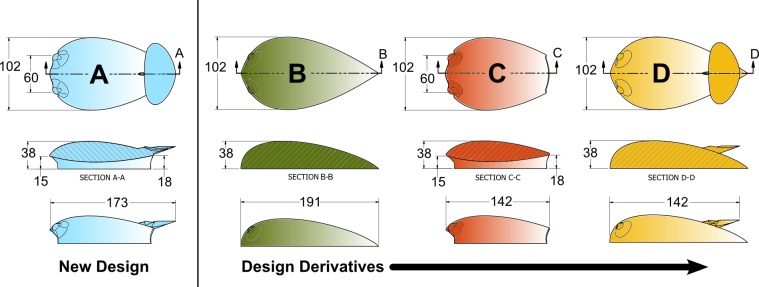
Tag housing designs in planform view (top row), longitudinal cross-section (middle row) and side view (bottom row). The longitudinal cross-sections are taken along the center line of the tags (marked A to D in the top row). The Model A design has a hydrodynamic body, channel and wing. Dimensions are in millimeters.

Here we extend the work in Shorter *et al*., and present a tag design that successfully uses flow control elements to reduce the net force acting on the body (Fig 1B). The design incorporates a hydrodynamic (streamlined) body, underbody channel, and inverted wing to reduce the net hydrodynamic force created by the tag. Further, we present a series of models that were used to compare these design features in simulation to clarify the contribution of individual components to overall force reduction, Fig 2. Such analysis enabled a systematic approach to the design process and allowed for a careful examination of the tradeoffs between design elements. Our simulation results indicate that the balanced use of a hydrodynamic shape, underbody channel, and wing has the potential to significantly reduce overall hydrodynamic loading on the animal. In support of the simulations, experimental measurements of the flow around the final design were conducted using particle image velocimetry (PIV). These results confirm the trends predicted in the simulations, helping to validate the simulated results.

## Methods

### Tag design

Bernoulli’s equation states that the sum of the local static pressure (*P*) and dynamic pressure ($\frac{1}{2}\rho V^{2}$) are constant along a streamline

$$P+\frac{1}{2}\rho V^{2}=C$$ (1)

where *ρ* is the density of the fluid and *V* is the speed of the fluid. Fluid moving quickly over a certain region of a body–but outside of the boundary layer–will result in a low pressure region [19]. Conversely, fluid moving slowly will result in a high pressure region. A bluff body immersed in a moving fluid forces the incoming flow to slow down at the surface, creating areas of high pressure that result in drag generating pressure differentials in the direction of the flow. Streamlined bodies reduce drag by redirecting flow around the surface in a controlled manner. If the streamlined body is also axisymmetric the flow around the body will be uniform, resulting in no net lift forces (e.g. no forces acting in a flow-normal direction). In contrast, non-axisymmetric bodies, such as a tag attached to an animal, create unbalanced pressure differentials that result in a net lift force acting on the body [18]. Road vehicles and racing cars, for example, generate lift forces that result in a reduced contact force with the road as they are driven [16–18,20]. The reduced downward force on racing cars can be counteracted by the use of wings, body shape, and special underbody designs. In an analogy to vehicle body shape designs that modify net vertical force, our objective here is to minimize the force that the tag exerts on its contact surface.

Functional tag components such as the electronic hardware, sensors, and battery along with the attachment mechanism and the floatation unit required for recovery create a fixed payload that limits the possible size and shape of the tag housing (see Shorter *et al*., (2014) for more details on these constraints). Simulations and experimental measurements of the forces generated by a suction cup attached tag with internal volume of 192 ml (called here Model 0, Fig 1A, and modeled on a recent version of the DTAG [21]) served as a point of reference for the designs presented in this work. Although a specific set of components were used to constrain the housing designs, the interpretations of the results are widely applicable to tags for aquatic animals. The work presented in Shorter *et al*. (2014) indicated that drag created by the Model 0 design could be reduced by modifying bluff design features present at the flow interface (e.g. the suction cups and the shape of the tag), and that large lift forces could be reduced by eliminating the asymmetric flow above and below the tag. To that end we examine how a hydrodynamic flow screen, an underbody channel, and a wing condition the fluid velocity, and resulting surface pressures, to reduce the net hydrodynamic loading on the tag (Model A, Fig 2).

The basic body shape and volume of the half-teardrop were constrained by the volume of the Model 0 tag. Additionally, it was assumed that the tag could be placed directly on the animal, in-line with the flow, and away from fins or body features that would obstruct the flow around the animal. The animal’s body serves as the bottom of the channel, and to perform optimally, the tag housing must be flush to the animal’s body to ensure that fluid is only allowed to travel through the channel. While the Model 0 tag is secured to the animal using suction cups, a combination of attachment mechanisms could be used to secure the base of the Model A housing to the attachment surface. This includes suction cups and/or adhesives depending on the surface (e.g. skin vs fur). Autodesk Inventor 2013 (Autodesk Inc San Rafael, CA USA) was used to create the 3D models of the tag geometry, CFD simulations were computed using the SolidWorks 2012 and 2013 Flow Simulation package (Dassault Systemes, Solid Works Corporation).

#### Channel design

Building on the work presented in Shorter *et*. *al*. (2014) the underbody was shaped as a Venturi channel with three main design parameters: the inlet shape, the location of the minimum cross-section, and the outlet shape [18]. The inlet serves as a collector, smoothly accelerating the fluid toward the minimum cross-sectional area. The flow velocity peaks at the location of the minimum cross-sectional area, resulting in minimum pressure. Downstream from the minimum cross-section, the flow slows down and pressure grows as the channel increases in width and height. The aft part of the channel was designed to ensure that static pressure at the outlet was close to the undisturbed pressure of the flow outside the channel. This reduced drag by eliminating pressure differences between the forward and aft surfaces of the tag.

The practical implementation of the Venturi channel involved considering several geometric constraints created by the mechanical elements of the tag. The height and width of the inlet were limited by internal volume constraints (i.e. room for the electronics) and the location of the suction cups used for attachment. The height of the inlet was made as large as possible to minimize the mass flow rate loss due to the momentum deficit close to the animal skin (i.e. due to boundary layer ingestion). The inlet of the channel was designed with a sharp leading edge to reduce flow damming in front of the housing, while favoring flow separation within the channel that results in low pressure to reduce lift in large off-axis flow angles. The outlet was designed to merge smoothly with the tear-drop shape of the outer shell, while maintaining large cross-sectional areas to guarantee pressure recovery. An iterative approach with feedback from CFD simulations was used to design the minimum cross-sectional area of the channel. The minimum channel width, height, and longitudinal location were modified prior to each simulation until smooth flow was observed throughout. This tuning process for the channel geometry involved about ten iterations and resulted in the final shape shown in Fig 2.

#### Wing design

**Fig 3:**
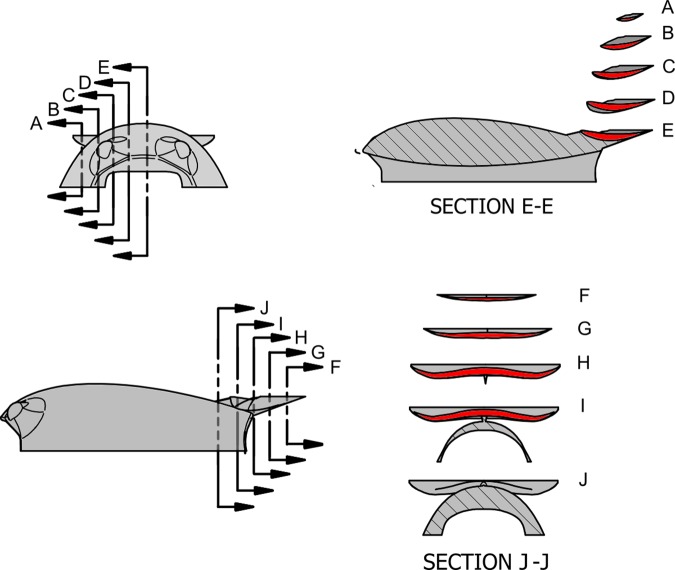
Detailed cross sectional views of the wing design. The cross sectional segment is highlighted in red. Top left: frontal view with section locations indicated. Top right: sections taken from the frontal view. Bottom left: side elevation with section locations indicated. Bottom right: sections taken from the side elevation.

Given the geometric constraints created by the tag suction cups and electronics, it was particularly challenging to compensate for the lift force acting on the body with only an underbody channel. In aeronautics, inverted wings are an efficient means of reducing the lift acting on bluff bodies. Redirecting the incoming flow up and away from the body can result in a net downward force [17–18]. Typically, a wing is characterized by large values of the lift-to-drag ratio, as opposed to spoilers which create moderate lift, but also large values of drag. For such a reason, a wing was preferred over a spoiler. The design of the wing involved selecting geometric parameters defining the wing span, wing surface, and the angle of attack with respect to the flow. Additionally, constraints on the wing span and wing height were set based on the tag geometry: the maximum wing span was set smaller than the maximum width of the tag, and the maximum wing height was set smaller than the maximum height of the body. Given such constraints, the wing position was fixed in the proximity of the underbody channel outlet, close to the surface of the outer shell. A single-pylon with a symmetric airfoil cross section was used to mount the wing to the body (Figs 2 and 3). This design was chosen to simplify fabrication, avoid recirculation around the pylons in off-axis flow, and to avoid large yawing moments created in side flow.

The incoming flow on the wing is heavily affected by the design of the tag body. To prevent a reduction in downward lift or an increased drag penalty, the wing was designed to closely follow the onset angle of the incoming flow and avoid flow separation. Additionally, induced drag created by the wing was reduced by keeping the spanwise distribution of the local angle of attack constant and by shaping the wing planform in an elliptical manner [16, 18, 22]. As with the channel, an iterative simulation based approach was used to finalize the wing design. During the design of the wing, eight stations were selected along the wing half-span, each set with an initial value for the incidence angle. The wing chord distribution along the span was set to obtain an elliptical planform. The swept wing geometry was then created by spline-interpolation through the wing stations, resulting in a smooth, twisted, and tapered wing. Finally, CFD simulations were run to evaluate the wing geometry. At each step, the CFD results were analyzed and flow separation regions (stall) were localized underneath the wing. The incidence angles of the wing stations were modified iteratively to achieve attached flow and downward lift, while observing the variations in added induced drag created by the wing. In order to achieve a desirable elliptical lift distribution along the span the angle of attack of the wing with respect to the local onset flow was kept constant. Detailed cross sections of the final wing shape are shown in Fig 3.

### Hydrodynamic loading

In general, the forces acting on a bluff body in a moving fluid, such as a tag attached to a swimming animal, are characterized by lift (${\overset{´}{F}}_{L}$), drag (${\overset{´}{F}}_{D}$), and side (${\overset{´}{F}}_{S}$) forces. Here, the lift force acts perpendicular to the attachment surface, the drag force acts along the flow direction and the side force is perpendicular to the plane formed by the drag and lift axes. The total hydrodynamic force $\overset{´}{F}$ acting on the body is defined as the vector summation of the component forces.

$$\overset{´}{F}={\overset{´}{F}}_{L}+{\overset{´}{F}}_{D}+{\overset{´}{F}}_{S}$$ (2)

To facilitate a comparison between tag designs, both those presented here and in the literature, the hydrodynamic forces are nondimensionalized to obtain force coefficients [17–18].

$$C_{L}=\frac{L}{0.5\rho V_{\infty }{}^{2}A_{ref}}$$ (3)

$$C_{D}=\frac{D}{0.5\rho V_{\infty }{}^{2}A_{ref}}$$ (4)

Where *A*_*ref*_ is the projected frontal area of the tag perpendicular to the direction of the flow, *ρ* is the density of the fluid, and *V*_∞_ is the free stream velocity. Additionally, the hydrodynamic efficiency (*E*) of the tag design is defined as the ratio of the dominant force coefficients where large positive values of *E* indicate large lift forces with respect to the drag generated by the body.

$$E=\frac{C_{L}}{C_{D}}$$ (5)

To eliminate dependence on the free-stream velocity, experimental and simulation data is used to calculate the coefficient of pressure (*Cp*) which is a dimensionless number that represents the relative pressure in the flow field.

$$Cp=1-{\left(\frac{V_{local}}{V_{\infty }}\right)}^{2}$$ (6)

With local velocity defined as,

$$V_{local}=\sqrt{u^{2}+v^{2}}.$$ (7)

Where *u* is the stream-wise velocity and *v* is the velocity perpendicular to the surface.

### Computational fluid dynamics simulations

**Fig 4:**
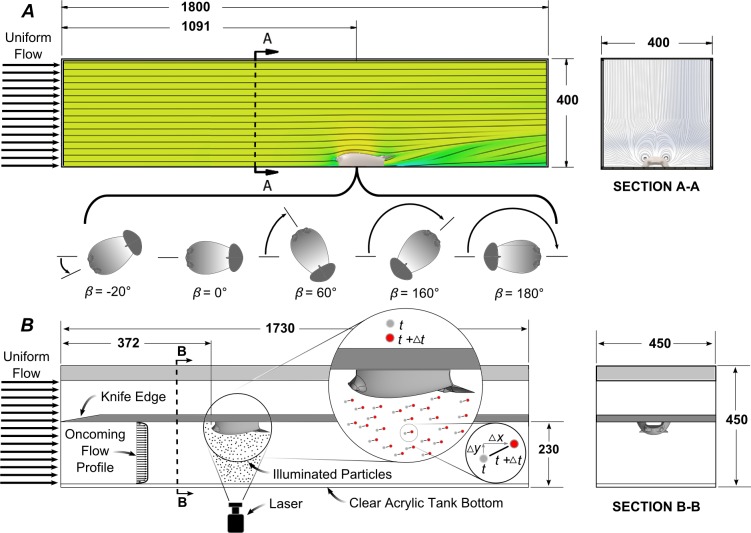
**A)** Illustration of the computational domain used for the simulations of all models. Streamlines from a representative simulation are also included in the figure. Representative orientations, *β*, of the tag in plan view during the examination of off-axis flow are shown below panel A. **B)** Illustration of the experimental PIV setup used to measure the flow around the Model A design. The figure inset presents how the illuminated particles are used to measure the velocity of the fluid. The fluid flow in both cases is from left to right, and dimensions are in millimeters.

An analysis of the hydrodynamic forces and moments created by the geometry of the tag designs were conducted using computational fluid dynamics (CFD) analysis. Computations were performed over a range of flow velocities, V_∞_ = 1–6 m/s to span the sustainable swimming speeds of a number of animals [13]. Additionally, the forces generated on the Tag A design were simulated in constant 5.6 m/s offaxis flow (*β* = 0°–180°, see Fig 4A for definition of the off-axis flow angle) to model the impact of poor attachment orientations in which the tag is rotated with respect to the longitudinal axis of the animal. The constant 5.6 m/s flow was selected to facilitate comparisons with the simulation and experimental results presented in Shorter *et al*. (2014). The constant flow speed and flat plate geometry of the attachment surface were simplifications made with respect to the real geometries to facilitate the simulations. However, these simulations enable the investigation into relative changes created by different flow control features. A second order polynomial was fit to the results of the point wise simulation using linear least squares, and used to provide a functional approximation of the forces acting on the tag body at flow velocities not directly run during the CFD simulations.

$$F_{fit}\left(v\right)=p_{1}v^{2}+p_{2}v+p_{3}$$ (8)

Where *p*_1_, *p*_2_ *and p*_3_ are the coefficients for the polynomial fit.

As in Shorter *et al*. (2014), simulations were performed in a computational domain (400 mm wide, 400 mm high and 1,800 mm long, Fig 4A). The flow solver computed the Favre-Averaged Navier-Stokes equations by closing the set of equations with a *k-ε* turbulence model. The turbulence parameters used here include a turbulence intensity of 0.5% and turbulence length of 5 cm. A no-slip, no roughness boundary condition was applied to the attachment surface (i.e., the animal's skin surface), and the domain inlet had a uniform velocity distribution. Free-slip boundary conditions were used at the domain outlet, ceiling, and side walls. Convergence of results was based upon lift, drag and side force steadiness. The mesh parameters and computational equipment used in Shorter *et al*. (2014) were also used here and more details about the selection of the parameters can be found in that work. The average solution time was approximately 2 h for the variable speed simulations and approximately 6 h for the variable orientation simulations.

### Experimental particle image velocimetry setup

Flow over a 3D model of the tag prototype was visualized in a temperature controlled recirculating flume (Engineering Laboratory Design, Inc., freshwater, 20 C) using particle image velocimetry, PIV [23,24]. The 3D model was made out of ABS plastic using a Dimension Elite 3D printer (Stratasys, Ltd, Eden Prairie, Minnesota, USA) with a layer resolution of 178 microns. The model was polished after manufacture to present a smooth surface, Fig 4B. The test section of the flume was 1730 mm in the streamwise direction, 450 mm in the cross-stream direction, and 450 mm in depth. The Model A tag was mounted on the downward facing side of a flat plate suspended horizontally in the flume test section. The plate spanned the entire length and width of the test section and its bottom surface was 230 mm above the bottom of the flume. The leading edge of the plate was machined to a 10° knife edge with the slanted section upward to control the formation of the boundary layer at the start of the plate. The tag was centered in the cross-stream direction and the front end of the tag was 372 mm from the leading edge of the flat plate (i.e., the inlet of the test section). The freestream velocity of the flow in the test section was 1.49 m s^-1^.

A laser sheet (Firefly, Oxford Lasers, 808 nm) 1 mm in thickness was oriented in the streamwise direction along the centerline of the tag. It illuminated silvered glass spheres (Potter Industries, SH400S20, mean radius 10μm) that were added to the flow. The particles were imaged with a monochrome digital camera (Fastcam SA3, Photron, resolution 1024 x 1024, 50 mm Nikon lens) oriented perpendicular to the light sheet. The field of view was 134 mm x 134 mm. The camera acquired frames at 60 Hz and the laser was strobed to generate exposures 0.5 ms apart, resulting in a flow field visualization rate of 30 Hz. Velocity fields (128 x 128 grid) were calculated using a multipass 2D FFT approach (DaVis 8.2, La Vision, Inc., 16 x 16 pixel subwindows, 50% overlap) and time-averaged over 5 s (i.e., averaging 150 flow characterizations). A robotic positioning system was used to move the camera and laser known distances in the streamwise direction so that a mosaic of the flow over a streamwise range of 680 mm could be constructed beginning at a distance 61 mm upstream of the front of the tag. The cross-stream velocity was not measured in the experiment and the local velocity was approximated as using Eq 7. In order to compare values between experiment and simulation, *Cp* data were normalized using the freestream velocity. A map of the differences was created by calculating the difference between values at each point on the grid,

$$C_{pdiff}=C_{psim}-C_{pexpt}.$$ (9)

The mean difference in the magnitude of the coefficients of pressure, *mean*(|*C*_*pdiff*_|), was calculated to provide an overall measure of agreement. A value for the mean difference in the magnitude of the coefficients of pressure close to 0 indicates good agreement between simulation and experiment.

## Results

### Simulation results for variable inline velocity

**Fig 5:**
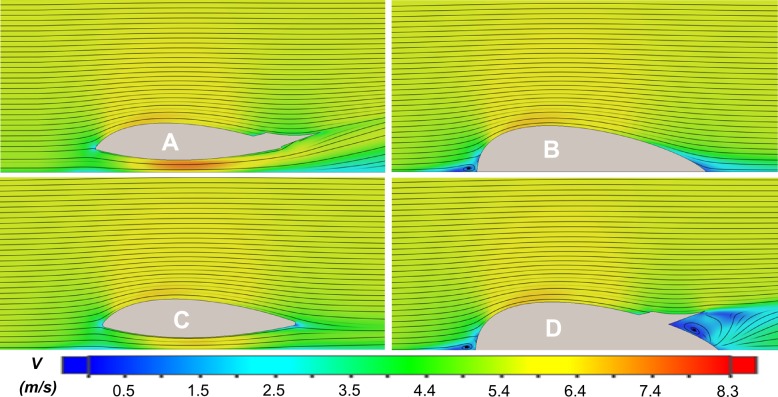
Longitudinal cross-section views in 5.6 m/s inline flow with a color map corresponding to the speed of the fluid flow (*V*). Areas of low speed are shown in blue, while areas of high speed are shown in red.

**Fig 6:**
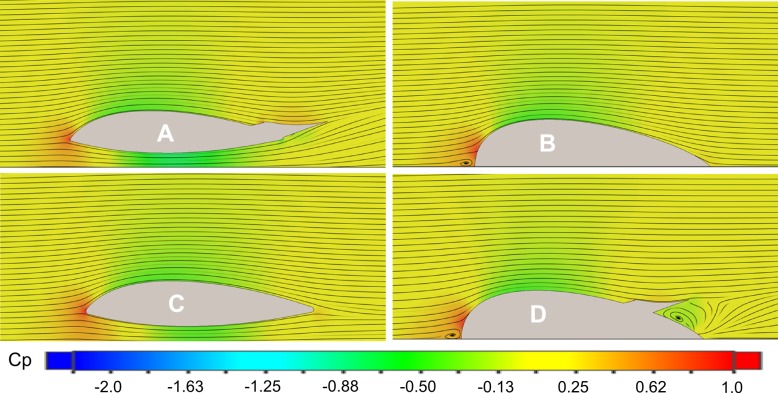
Longitudinal cross-section views in 5.6 m/s inline flow with a color map corresponding to the coefficient of pressure (*Cp*). Areas of low pressure are shown in green, while areas of high pressure are shown in red.

The effects of the different design elements were clearly demonstrated by the CFD simulations in variable inline flow. Representative images of the four tag designs detailing velocity, pressure and the streamlines of the fluid for inline 5.6 m/s flow are shown in Figs 5 and 6. Beginning with the design derivatives, the flow around the Model B tag remains attached to the surface as the hydrodynamic form redirects fluid around the body. The airfoil-like shape accelerates the fluid over the top of the tag creating the lift-generating area of low pressure (Figs 5 and 6; top right). The flow interface at the front of the Model B design creates some fluid damming, indicated by the reduction in fluid velocity and corresponding increase in fluid pressure that results in drag-creating pressure differentials.

The addition of the channel in the Model C design allowed fluid to flow under the tag. The shape of the channel also increased the speed of the fluid to nearly the free stream speed. However, the Model C shape still created a larger area of low pressure above the tag than in the channel, resulting in a net lift force (Figs 5 and 6; bottom left). The wing on the Model D design redirects flow away from the tag and slows the fluid down over the wing but also creates an area of recirculating separated flow below the mid-span of the wing that results in an area of low pressure. In fact, the flow in this region is subject to a rapid area expansion created by the lower surface of the wing and the top of the housing. This expansion does not allow a proper pressure recovery and results in flow separation. The high pressure at the front of the tag created by the fluid damming combined with the low pressure below the wing results in an increased drag force (Figs 5 and 6; bottom right).

Combining the channel and the wing in the Model A design enhances the contribution of the channel and creates a net downforce acting on the body. The flow under the tag now reaches speeds around 8 m/s creating a large area of low pressure (Figs 5 and 6; top left). Moreover, the synergy of the wing and channel allows the flow to travel at a higher velocity at the back of the tag, when compared with the models using only one of the two features (C and D). This effect is responsible for an overall reduction in lift, but also creates a low pressure region that extends farther along the Venturi channel, which marginally increases the balance of the net hydrodynamic drag. Table 1 illustrates how the different design elements contribute to the net drag and lift on the Model A design.

**Table 1:** Component and net tag force contributions from the individual flow control elements for the Model A design in 5.6 m/s flow.

*Model A*	*Side (N)*	*Lift (N)*	*Drag (N)*	*Net (N)*
**Body**	0.30	51.60	5.60	51.90
**Channel**	0.03	-36.40	3.50	36.50
**Wing**	0.05	-30.80	1.10	30.90
**Assembly**	0.40	-15.60	10.20	18.70

**Fig 7:**
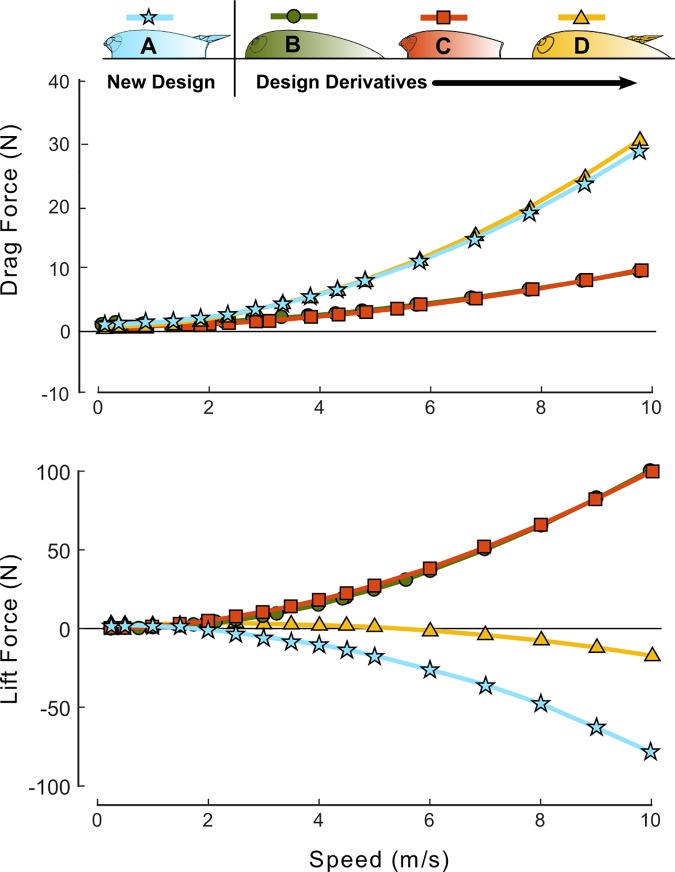
Net drag and lift forces for each tag design in variable-speed inline flow. At the higher speeds the wings generates a net downward force but also more drag. Less drag is created without the wing but the lift forces increase greatly. Second order polynomial fits (solid lines) were used to interpolate between simulation data points. Fitting parameters and *R*^*2*^ values are presented in Table 3.

The CFD simulations were used to calculate the net drag and lift forces acting on the tag designs as a function of flow speed (Fig 7). Symmetric flow around the models was maintained over the range of selected speeds for the variable inline flow simulations, resulting in negligible side forces acting on the bodies. As such, only the net drag and lift forces were used to make comparisons between designs in Fig 7 and Table 2. The parameters for the second order polynomial fits shown in Fig 7 are presented in Table 3. The new design and the design derivatives have comparable performance at low flow speeds (*S* < 1 m/s). As the speed of the flow increases, the drag generated by the designs without the wing remains low, increasing to only around 9 N in 10 m/s flow, while the designs with the wing create three times more drag (~30 N). In contrast, the designs without the wing create upwards of 100 N of lift at peak speeds, compared to the downward force (negative lift) created by the models with the wing as shown in both Fig 7 and Table 2. Overall, adding the wing increases the drag coefficient (*Cd*) and decreases the lift coefficient (*Cl*). The combined model has a negative efficiency (*E*) indicating that the Model A design creates a net downforce. Summary data from the simulations are provided in the supporting information (S1 File).

**Table 2:** Comparisons of peak forces and dimensionless force coefficients for the tag designs in 6 m/s aligned flow. Adding the wing increases the drag coefficient (*Cd*) but decreases the lift coefficient (*Cl*). This results in a negative efficiency (*E*) indicating that the Model A design creates a net downforce.

*Tag Design*	*Speed (m/s)*	*A*_*ref*_ *(m*^*2*^*)*	*Drag (N)*	*Lift (N)*	*Net (N)*	*Cd*	*Cl*	*E*
**Model A**	6	0.0024	10.13	-28.56	30.30	0.23	-0.65	-2.82
Model B	6	0.0028	3.28	38.53	38.67	0.06	0.74	11.74
Model C	6	0.0024	3.48	37.00	37.16	0.08	0.84	10.64
Model D	6	0.0028	10.93	-3.68	11.53	0.21	-0.07	-0.34

**Table 3:** Coefficients and *R*^*2*^ values for the second order polynomial fits presented in Fig 7.

Model	Force	*p*_*1*_	*p*_*2*_	*p*_*3*_	*R*^*2*^
**A**	Drag	0.2918	-0.06432	0.03022	1.00
	Lift	-0.7584	-0.1952	0.1267	0.99
**B**	Drag	0.07913	0.07119	-0.02623	0.99
	Lift	0.9009	1.053	-0.4077	0.99
**C**	Drag	0.08716	0.04477	-0.00208	1.00
	Lift	1.035	-0.1429	-0.3607	0.99
**D**	Drag	0.2918	0.07907	-0.05286	1.00
	Lift	-0.3327	1.46	-0.6659	0.99

### Simulation results in off-axis flow

**Fig 8:**
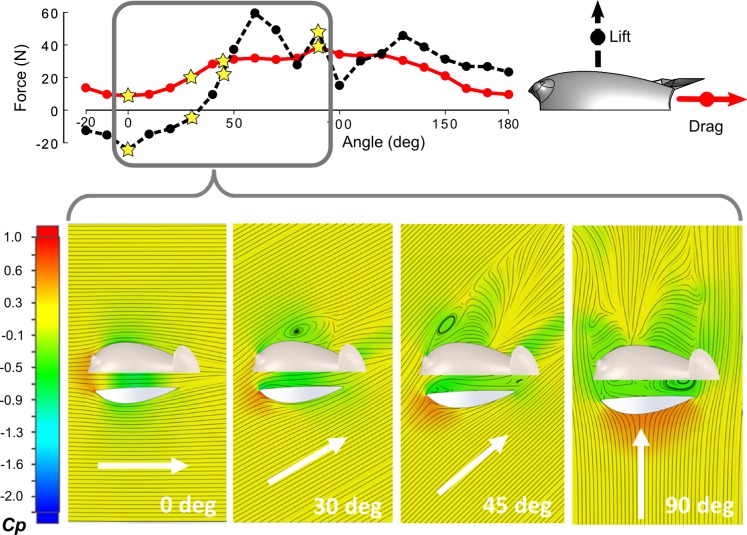
Net drag and lift forces for the Model A tag in off-axis 5.6 m/s flow. Simulation results for four orientations (0°, 30°, 45° *and* 90°) with the corresponding stream lines and color map of the resulting pressure field acting on a transverse plane parallel to the attachment surface.

Simulations of the Model A tag in off-axis flow were conducted to examine the effect of tag misalignment on the performance of the flow control elements, Fig 8. In general, tag misalignment had a greater negative effect on the magnitude of the lift force acting on the tag. For misalignments of *β* < 30°, the flow remains fully attached to the channel walls and the channel/wing continues to create a low pressure region within the channel. At these small yaw angles the lift acting on the tag is still negative, but the negative lift force is decreasing while the drag has begun to increase. At *β* > 30°, the flow becomes partially detached on the downstream side of the housing and in the channel. As the angle of the flow to the tag continues to increase, the fluid becomes increasingly separated from the body, with a larger area of recirculating flow now present in the channel. At *β* = 90°, the flow through the underbody channel is completely recirculating. The coefficient of pressure *Cp* however, is low throughout the channel due to the sharp separation of the flow at the edges of the channel (see Fig 8). This ensures a moderate suction of the tag on the animal skin, even when the flow is stalled within the channel. As the tag is rotated past 90° the drag force begins to decrease, but the lift force remains above 20 N in the 5.6 m/s flow. Summary data from the simulations are provided in the supporting information (S1 File).

### Experimental PIV results

**Fig 9:**
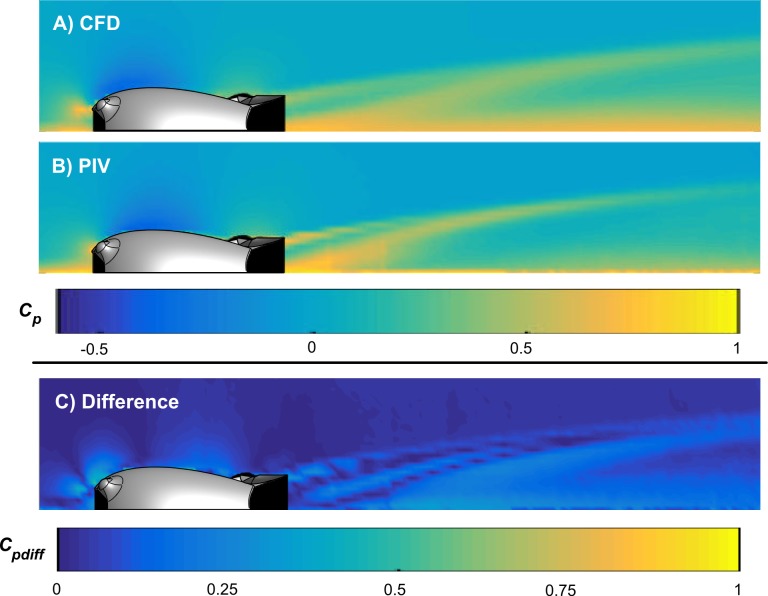
Comparison of simulated **(A)** and experimental **(B)** coefficients of pressure (***C***_***p***_) acting on the Model A tag. The experimental measurements were made using particle image velocimetry and compare well to the simulated results. This agreement is highlighted by the small differences in coefficients of pressure **(*C***_***pdiff***_**)** presented in subplot **(C).** The black mask shown in (A), (B) and (C) identify areas that were shadowed in the PIV measurements.

Experimental measurements of the flow around a solid 3D prototype of the Model A design were made using particle image velocimetry to validate the simulation results. Fig 9 shows the normalized fluid pressure around the Model A tag calculated from both simulated (Fig 9A) and experimental (Fig 9B) data. A map of the differences in calculated coefficients of pressure, *Cpdiff*, is presented in Fig 9C. The mean difference in the magnitude of the difference in coefficients of pressure was 0.08, indicating excellent agreement between the simulated and experimental results. Expected features such as a negative coefficient of pressure on the top and a positive value at the front of the tag are present. More significantly, the similarities in the structure of the wake, with a central jet emanating at an angle out of the channel, and a region of slower flow just below the wing demonstrate the potential effectiveness of the flow control elements. While these similarities are striking, there are differences clearly visible between the two Figs. Two examples are the more forward-extending region of low pressure above the tag in the simulation, and the differences in the pressure around the jet emanating from the channel due to slight differences in the shape of the wake. Also, the boundary layer downstream of the tag is thicker in the simulation. These differences could be the result of the walls in the PIV flume that were not modeled in the simulation. These walls form a water channel, and the blockage due to the Model A tag may result in fluid flow not captured by the simulations. The thin wing of the 3D printed tag may also have deformed slightly under hydrodynamic loading, which would not be modeled by the rigid tag in the simulation. Summary data for the experimental results are provided in the supporting information (S2 File).

## Discussion

This work explores the performance tradeoffs in adding flow control elements to a hydrodynamic tag design using a simplified simulation environment, and presents experimental data to validate the results. The new tag design (Model A) presented here incorporates a low-drag half-teardrop shape and flow control elements to reduce the net loading on the housing. The Model A design builds on the work presented in Shorter *et al*. (2014), but with the important distinction that the simulation and experimental results for the Model A tag indicate that the flow control elements will result in a reduction to the net loading on the tag, unlike the channel-only design presented in Shorter *et al*. (2014).

The investigation into the performance tradeoffs of the individual design features of the Model A tag indicates that the hydrodynamic housing alone effectively reduced the drag force to just 3 N in 5.6 m/s flow (Model B; Figs 5 and 6). In comparison, simulations of a similar sized tag design without these features (Model 0, Fig 1A) in Shorter *et al*. (2014) indicated four times more drag (13 N). Thus, drag is reduced effectively by adopting a smooth-walled teardrop shape for the tag hull. However, the accelerated fluid flow over the top of the Model B housing created a lift force of ~35 N, comparable to that of Model 0 (~36 N). The Venturi channel improved the symmetry of flow above and below the tag (Model B; Figs 5 and 6). The channel design successfully accelerated flow under the tag body without increasing drag loading. However, geometric constraints imposed by the suction cups and tag electronics limited the channel size and the amount of water moving under the tag. This resulted in a pressure within the channel that was greater than the pressure above the Model C design, preventing the cancellation of lift forces with the channel alone (Model C; Fig 7). The fit between the bottom of the tag and the skin interface is also an important practical design consideration. Any slow-moving fluid between the housing and skin on the sides of the channel will reduce the performance of the design feature. While a perfect fit between the tag and skin is not likely, a flexible housing could be used to help the tag conform to the contoured surface.

The wing, like a spoiler on a car, created a force to oppose lift generated by the tag body using a combination of negative lift generated by the wing shape and redirection of flow up and away from the tag. The wing not only reduced the lift acting on the tag, but created a net downward force at higher speeds. However, the simulated 4 N reduction in lift at 6 m/s was offset by a threefold increase in drag acting on the tag (Model D; Fig 7). This drag penalty has been observed in other systems [16, 18], and can be reduced by modifying the design of the wing tips using winglets or endplates (McCormick, 1995; Barnard, 2009; Katz, 2003). However, from a practical standpoint, the wing represents an appendage which may be subject to unwanted collisions or entanglements when the tag is on the animal. This risk could be reduced by creating a weak link at the point of attachment with the tag allowing the wing to be shed from the tag body in the event of entanglement.

The use of the channel and wing in combination with the hydrodynamic housing (Model A) was more effective than either individual element at reducing the lift force. The co-design of these two elements increased the downforce by ~25 N at 6 m/s and ~62 N at 10 m/s over the wing alone. In the combined design, the wing redirects flow up and away from the tag, and the resulting change in momentum creates a downforce on the tag, (Fig 9). The visible central jet emanating at an angle out of the channel in the PIV results clearly demonstrates this effect experimentally (Fig 9B). The wing shape creates high pressure on the top surface and low pressure below the wing resulting in additional downforce (Fig 6A). The low pressure created under the wing also increases the speed of the flow through the channel, increasing the mass flow rate, decreasing the pressure in the channel and further increasing the net downforce acting on the Model A tag (Fig 5A).

The Model A design was robust to some flow misalignment as would arise if the tag were not aligned longitudinally on the animal's body. The channel/wing system continued to create a downforce until the off-axis flow exceeded 45 degrees. The forces increased to peak values of around 30 N of drag in 90 deg off-axis flow and around 60 N of lift in 60 deg off axis flow. While significantly larger than the forces generated in the ideal orientation, these values are comparable to the forces created by the Model 0 design in the same set of off-axis flow conditions presented in Shorter *et al*. (2014). As such, the simulation results indicate that the performance benefits of the Model A design have the potential to translate to the field where an ideal tag alignment is often not possible. The off-axis flow analysis highlights the importance of the synergy between the channel and the inverted wing. In fact, it would be challenging to achieve any reduction in lift force in misaligned flow by means of an inverted wing only. Throughout the off-axis flow simulations, the features that produce downforce shift from the channel and wing (lo*w β*), to primarily the channel (moderate *β*), and finally to the channel only (high *β*).

While promising, the findings presented in this work are subject to several limitations. A fully functioning tag prototype utilizing the Model A design has yet to be fabricated as only 3D printed representations of the shapes were made for the experimental work. Reliable manufacture and field deployment of a complete tag may require some modification of the idealized designs. An additional issue is that the CFD simulations do not account for the complicated flow around a swimming animal, or the boundary layer thickness at a typical tag placement location. Enhanced CFD environments with considerably larger meshes and correspondingly longer computation time will need to be developed for these features to be incorporated. But although the values computed here may not accurately predict the forces in a real situation, the simulation results may still be valuable for comparing the relative performance of the flow control features. Future work will involve the fabrication of working prototypes based on the Model A tag design, and the experimental evaluation of the designs using controlled swimming trials with dolphins in managed environments. Additionally, we plan to improve the simulation environment by including 3D models of cetaceans that can be posed in postures observed during swimming. These modeling efforts will provide the ground work for future models that will better capture the dynamics of swimming.

This work was motivated by the observation that the net force induced by the tag may perturb the biomechanics of swimming in the host animal. A large tag-induced force is likely to require additional positive work and/or a modified gait in response. Previous studies have demonstrated that hydrodynamic tags create a lift force that is significantly larger than the drag force at fast swimming speeds suggesting that design efforts to reduce lift rather than drag are needed to reduce the total force (Shorter *et al*., 2014). Shorter *et al*. also suggested that flow control elements such as channels and spoilers could be used to reduce these lift forces. In this work, the hydrodynamic housing, channel and wing, used together, achieved a 30% reduction in net loading when compared to a similar-sized but conventionally-shaped housing in 10 m/s flow. Shorter *et al*. (2014) tested the ability of the suction cups used to secure the Model 0 tag to resist both normal and shear loading using a dolphin cadaver. In that work, the suction cups were able to resist shear loads of up to 94 N before the cups began to slide on the attachment surface, and normal loading of up to 181 N before the cups became detached from the cadaver’s skin. If those same cups were used to secure the Model A design presented in this work, the polynomial fit of the drag simulation data predicts that the animal would have to reach speeds approaching 18 m/s (40 mph) before the drag force would reach that threshold. But unlike the tag designs presented in Shorter *et al*. (2014), the interpolated simulation data predicts that the Model A design would generate ~250 N of negative lift pushing the tag on to the animal and never exceeding the threshold for failure during normal loading. This negative lift force would press the tag against the animal’s body instead of lifting it away, and may serve to provide additional resistance to the sliding mode of attachment failure created by the drag loading.

Despite the overall reduction in net force, along with the generation of a negative lift force, the addition of a wing increased the drag of the Model A tag by a factor of three compared to the design derivatives without the wing. Although increased drag loading has been shown to impact aquatic animals [6, 7], the effect of a force acting perpendicular to the animal’s body has not been explored explicitly; although it was almost certainly present in the tags used in these studies. Depending on the animal’s size, orientation and swimming speed the lift force from the tag could potentially affect the dynamic force balance of the animal possibly calling for a modified gait or changing the effectiveness of gliding with a resultant change in efficiency of locomotion [25, 26, 27]. To examine this, it will be important to determine the magnitude of the tag forces relative to the forces involved in natural swimming but scant information on these forces is currently available for most marine mammals [28–30]. Further, no matter what the attachment method, the forces generated by the tag must be balanced by forces on the skin at the attachment points. Reducing the total force acting on the tag will reduce the risk of local tissue trauma enabling longer and more reliable tag attachments. Overall, the reduction of net loading on the animal has the potential to lessen any potential energetic penalty, reduce the potential impact of skin loading at the point of attachment and may also help to limit changes in animal behavior.

## Supporting information

S1 File Simulation results for Model A tag housing design and the design derivatives over a range of flow velocities (*V*_∞_ = 1–6 m/s) are included in the supplemental data file. Additionally, data from forces generated by the Model A design in constant 5.6 m/s off-axis flow (*β* = 0°–180°) are also provided.
(XLSX)

S2 File This supplemental file includes the experimental measurements and simulation data used to generate the normalized fluid pressure around the Model A design. These data were used to create the difference map between the coefficients of pressure (*Cpdiff*) that was used to compare experimental and simulation results. The units for the velocity fields are (m/s).
(ZIP)
